# Exploratory Dietary Approaches for Drug-Resistant Epilepsy Beyond Standard Ketogenic Diet and Fish Oil: A Systematic Review of Preliminary Clinical Evidence

**DOI:** 10.3390/neurolint18010009

**Published:** 2026-01-04

**Authors:** Xianghong Meng, Kequan Zhou

**Affiliations:** 1 Shenzhen University Medical School, Shenzhen University, Shenzhen 518060, China; dr_mengxh@szu.edu.cn; 2 Department of Neurosurgery, Shenzhen University General Hospital, Shenzhen University, Shenzhen 518055, China; 3 Department of Nutrition and Food Science, Wayne State University, Detroit, MI 48202, USA

**Keywords:** drug-resistant epilepsy, ketogenic diet modifications, probiotics, gut–brain axis, low glutamate diet, systematic review

## Abstract

**Background:** Standard ketogenic diets (KD) and fish oil have established efficacy for drug-resistant epilepsy (DRE), but adherence and variability remain challenging. **Objective:** The objective of this study is to provide the first systematic evaluation of clinical evidence for emerging dietary interventions for epilepsy—specifically those other than standard KD and fish oil—and to rigorously evaluate their effectiveness and certainty of evidence to address the current gap in dietary management literature. Unlike prior reviews focused on standard KD or carbohydrate-modified versions, this study is the first to synthesize evidence for “non-standard” interventions—including olive oil-based KDs, probiotics, and restrictive gluten/glutamate-free diets—which are typically excluded from traditional dietary meta-analyses. **Methods:** Following PRISMA 2020 guidelines, we searched PubMed, Web of Science, Cochrane, and Google Scholar up to March 2025. Randomized Controlled Trials (RCTs) and Non-Randomized Studies of Interventions (NRSIs) were included, with quality assessed using RoB 2 and ROBINS-I tools. **Results:** Eight studies (total *n* = 675) were identified, comprising 2 RCTs and 6 NRSIs. These included olive oil-based KDs (*n* = 1), probiotic/synbiotic supplementation (*n* = 2), medium-chain triglyceride (MCT) additions (*n* = 2), and gluten-free (*n* = 1) or glutamate-free (*n* = 1) diets. Evidence quality is generally low, with 75% of studies at high risk of bias. Preliminary responder rates reached 83.1% in uncontrolled olive oil-based KD studies, whereas the only RCT evaluating a low-glutamate diet showed no significant seizure reduction (*p* = 0.57). **Conclusion:** Evidence for emerging dietary interventions beyond standard KD is nascent and of low certainty. **Interpretation:** While preliminary signals exist for olive oil-based KDs and probiotics, current data are insufficient for clinical recommendation; this review identifies these as promising exploratory targets requiring validation through rigorous, blinded RCTs.

## 1. Introduction

Epilepsy affects more than 50 million people across the globe including both children and adults [1]. While antiepileptic drugs (AEDs) are available, about 30–40% of epilepsy turns into drug-resistant epilepsy (DRE) [2]. DRE poses a significant threat to quality of life and creates a heavy economic and social load on families and society. This clinical challenge has driven scientists to explore novel therapeutic avenues, including dietary interventions [3,4,5,6,7,8,9,10,11,12].

Since the 1920s, the ketogenic diet (KD) has developed and has quickly become a cornerstone dietary approach to manage DRE [3,4,5,6,10,13,14,15,16]. The KD, a high-fat, low-carbohydrate, and controlled protein diet, is thought to exert its anticonvulsant effects by, among other mechanisms, boosting the action of GABA nerve signals through reducing available oxaloacetate for the aspartate aminotransferase reaction [13,17,18,19]. GABA is a known brain chemical that slows nerve activity and helps stop seizures [20,21]. While the classic ketogenic diet (KD) has served as a therapeutic cornerstone since the 1920s, its long-term clinical utility is often hampered by poor palatability and restrictive protocols that lead to high attrition and low compliance [14]. Similarly, while omega-3 supplementation (fish oil) has shown anti-inflammatory potential, clinical results remain variable across different seizure types [3,7,22,23]. Clinical trials have shown the promise of KD and fish oil supplementation, two of the most popular dietary interventions for epilepsy [3,6,7,15,22,23,24]. The effectiveness and quality of evidence of these diets have been updated in recent systematic meta-analyses [9,15,25]. Consequently, there is an acute clinical need to investigate dietary solutions that are more tolerable and target novel pathophysiological pathways, such as the gut-microbiota-brain axis and metabolic fuel shifts.

These emerging interventions, often supported by mechanistic insights or strong animal data, have advanced into human clinical trials [11,24,26,27,28,29,30,31]. Notably, probiotics and symbiotics have been investigated in managing epilepsy by targeting the gut microbiota via the gut–brain axis [11,26,32,33]. Scientists have explored supplementation of branched amino acids (BCAAs) as an adjunct therapy for epilepsy [31]. Animal studies showed that BCAAs can promote brain GABA production by promoting glutamate dehydrogenase activity [34,35,36,37]. Additionally, alternative diets have been explored in animal and human studies [24,28,29,30]. Notably, the prevalence of celiac disease in patients with epilepsy is estimated to be as high as 2.5%, compared to approximately 1% in the general population [29]. This provides a strong clinical rationale for investigating gluten-free diets (GFD) in this sub-population, where epilepsy may manifest as a neurological symptom of systemic gluten sensitivity [38,39].

A critical gap in the literature exists regarding these emerging strategies. While the effectiveness and quality of evidence for standard KD, its carbohydrate-modified versions (e.g., Modified Atkins Diet), and fish oil supplementation have been extensively updated in recent systematic reviews and meta-analyses, the clinical effectiveness and safety of these other novel dietary strategies have not been systematically evaluated. Although these novel dietary strategies show promise and are supported by mechanistic insights and animal studies, their supporting evidence in humans consists of disparate studies with varied designs. Currently, there are no comprehensive reviews available to examine these specific human studies. This review is therefore crucial to synthesize the available evidence, rigorously assess its quality and certainty, and provide clear guidance for clinical practice and future research. This manuscript systematically reviews the preliminary clinical evidence for these emerging strategies, replacing broad categorical summaries with a deep, individual study analysis to evaluate their potential role in DRE management.

## 2. Method

### 2.1. Protocol and Search Strategy

The current systematic review strictly follows the Preferred Reporting Items for Systematic Reviews and Meta-Analyses (PRISMA) guidelines (Table S1 checklist) [40]. The protocol for this systematic review was registered with the International Platform of Registered Systematic Review and Meta-analysis Protocols (INPLASY) under the registration number INPLASY2025120105. All procedures were conducted in accordance with the pre-defined PICOS framework detailed in Section 2.2. Two reviewers independently conducted a comprehensive literature search in PubMed, Web of Science, the Cochrane Register of Studies, and Google Scholar, encompassing publications available up to March 2025. To identify ongoing or unpublished trials, we also searched ClinicalTrials.gov and the WHO International Clinical Trials Registry Platform (ICTRP). The search strategy utilized a combination of Medical Subject Headings (MeSH) terms (e.g., “Epilepsy”, “Seizures”) and keywords (“diet”, “dietary supplementation”, “probiotic”, “amino acids”, “proteins”, “oils.”). All retrieved pubs were compiled using Endnote software (20th version).

### 2.2. Inclusion and Exclusion Criteria

We applied the following Population, Intervention, Comparator, Outcome, and Study Design (PICOS) framework:

- Population: Humans of any age with a confirmed diagnosis of epilepsy.
- Intervention: Any dietary intervention other than a standard ketogenic diet (KD), modified Atkins diet, KD modified only by adjusting carbohydrate portions, or isolated fish oil/omega-3 fatty acid supplementation.
- Comparator: No intervention, placebo, usual diet, or an active dietary intervention.
- Outcomes: At least one outcome related to seizure control (e.g., seizure frequency, responder rate, e.g., ≥50% reduction in seizures], seizure freedom).
- Study Design: Randomized controlled trials (RCTs) and non-randomized studies of interventions (NRSIs), including quasi-experimental and pre-post studies.

We included studies of humans with confirmed epilepsy receiving any dietary intervention other than the standard KD, modified Atkins diet, or isolated omega-3 supplementation. Both RCTs and NRSIs were eligible. Case reports and animal studies were excluded to prioritize evidence from clinical trials and structured human interventions. Two reviewers (XM, KZ) screened titles, abstracts and full-text reports to determine eligibility, resolving any discrepancies through discussion.

### 2.3. Data Extraction

Data were extracted regarding study design, participant characteristics, intervention duration, and seizure frequency. Quality was assessed using the RoB 2 (for RCTs) and ROBINS-I (for NRSIs) tools.

### 2.4. Quality Assessment

The quality of the included studies was assessed using the Cochrane Handbook for Systematic Reviews of Interventions [41]. Two reviewers (XM, KZ) evaluated each study’s risk of bias. For RCTs, the Cochrane Risk of Bias 2 (RoB 2) tool was used. For NRSIs, the Risk Of Bias In Non-randomized Studies-of Interventions (ROBINS-I) tool was applied. Domains assessed included the randomization process, deviations from intended interventions, missing outcome data, measurement of outcomes, selection of reported results, and overall bias. Disagreements were resolved through consensus. A sensitivity analysis was planned, including only studies at low risk of bias, to assess the robustness of the findings.

### 2.5. Data Synthesis

A formal meta-analysis was not performed due to substantial clinical (diverse interventions and populations), methodological (different study designs), and statistical heterogeneity (I^2^ > 80% in preliminary analyses). Instead, a structured narrative synthesis was conducted with results grouped by the pre-specified intervention category.

## 3. Results

### 3.1. Study Selection and Characteristics

**Figure 1:**
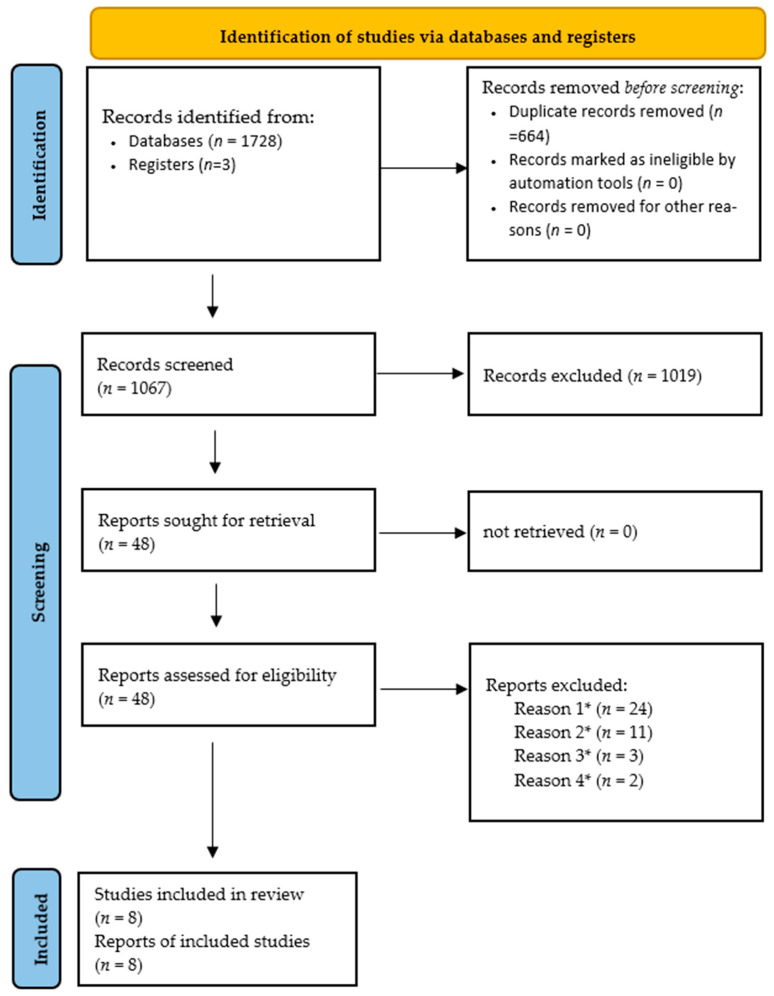
Study flow diagram. * Reason 1: Focused on standard Ketogenic Diet or carbohydrate-modified versions; Reason 2: Focused on Omega-3 fatty acid/fish oil supplementation; Reason 3: Case reports; Reason 4: Did not meet specific study scope/design.

Figure 1 presents the detailed search flow. Two authors independently conducted a comprehensive literature search across four databases and 3 additional sources. Following the removal of duplicates, 1728 unique records were screened based on title and abstract, leading to 48 full-text articles on human studies being assessed for eligibility. Of these, 40 articles were excluded for not meeting the specific scope of this review: 24 studies focused on conventional ketogenic diets or their carbohydrate-modified versions, 11 focused on omega-3 fatty acid (fish oil) supplementation, and 3 were case reports. The exclusion of these studies, for which systematic reviews already exist, was critical to isolating the evidence for the emerging interventions defined in this review’s objectives. Ultimately, 8 human trials met all inclusion criteria and were included in this systematic review [11,24,26,27,28,29,30,31]. The detailed study selection process is illustrated in the PRISMA 2020 flow diagram (Figure 1).

A total of 675 epilepsy patients were included across the eight eligible studies. All patients were on antiepileptic drugs (AEDs) during dietary interventions, establishing these approaches as adjunctive therapies. Intervention durations varied widely, from 1 month to 24 months.

The study designs were predominantly non-randomized. Only two studies were RCTs [27,30], while the remaining six were single-arm pre-post intervention studies or prospective cohort studies. The populations studied included both children and adults, with participant numbers ranging from a pilot study of 7 to a large prospective trial of 389. Further details on the study designs and populations are provided in Table 1.

### 3.2. Synthesis of Results (Qualitative Analysis of the 8 Included Studies)

Due to the significant clinical diversity among the interventions and high statistical heterogeneity (I^2^ > 80%), a formal meta-analysis was not performed. Instead, a structured narrative synthesis was conducted through a detailed, individual analysis of each included study to evaluate their specific potential in DRE management. This approach intentionally replaced broad categorical grouping with a qualitative assessment of individual results, focusing on primary efficacy outcomes such as seizure frequency, responder rates, and seizure freedom. By evaluating studies individually, the synthesis rigorously accounted for the unique risk of bias, varied participant demographics, and the preliminary nature of the evidence for each distinct dietary strategy.

1. BCAA Supplementation (Evangeliou 2009 [31]): This study (*n* = 17) investigated BCAAs as an adjunct to KD. While 18% of patients achieved seizure freedom, the pre-post design makes it impossible to distinguish the BCAA effect from the underlying KD efficacy.
2. MCT-based KD (Neal 2009 [30]): This high-quality RCT (*n* = 145) compared MCT-based KD directly to a classical KD. Finding no statistically significant difference in seizure frequency, it suggests that MCT protocols are an effective alternative but do not provide a superior seizure-control advantage.
3. Olive Oil-Based KD (Guzel 2019 [24]): This represents the largest study in our review (*n* = 389). It reported an 83.1% responder rate at 12 months. While encouraging, the lack of a control group and a 25.7% dropout rate limit the practical conclusion. The study suggests that Mediterranean-style fat sources may improve the long-term sustainability of the KD, but efficacy cannot be definitively attributed to olive oil without head-to-head RCT data against standard vegetable oils.
4. Probiotics (*Lactobacillus/Bifidobacterium*) (Gómez-Eguílaz 2018 [11]): This pilot study (*n* = 45) observed a 28.9% responder rate. While it establishes the gut–brain axis as a viable clinical target, the open-label design and self-reported seizure diaries introduce a high risk of bias, particularly regarding the placebo effect.
5. Synbiotics (Shariatmadari 2024 [26]): This quasi-experimental study (*n* = 30) reported a significant mean seizure reduction (*p* = 0.001). However, the 8-week duration is insufficient to determine long-term efficacy or potential shifts in the gut microbiome.
6. Supplemental MCT (Rasmussen 2023 [28]): This pilot study (*n* = 9) added MCT oil to a regular diet. While a 42% seizure reduction was reported, the sample size is critically low, essentially serving as a case series.
7. Gluten-Free Diet (Bashiri 2016 [29]): The 86% seizure freedom rate reported in this small NRSI (*n* = 7) is striking. However, all participants had confirmed celiac disease. The clinical takeaway is that GFD is highly effective only when systemic gluten sensitivity is the primary driver.
8. Low Glutamate Diet (Sarlo 2023 [27]): This non-blinded RCT (*n* = 33) is the only trial to specifically test a restrictive diet against a control group. The negative result (*p* = 0.57) suggests that dietary glutamate restriction may not be an effective monotherapy for pediatric DRE, despite its mechanistic popularity.

Given the high risk of bias identified, the efficacy results must be interpreted with extreme caution. The primary efficacy outcomes for all eight studies are summarized in Table 2.

## 4. Discussion

The central finding of this systematic review is the marked conflict between the plausible mechanisms of alternative diets and the current lack of high-quality clinical evidence. interventions such as probiotic supplementation targeting the gut–brain axis or BCAA additions targeting GABA synthesis are supported by robust animal models [32]. In a 2009 single-arm pre-post trial [31], BCAAs plus KD appeared promising, over 50% of participants (8 out of 17) experienced a ≥50% reduction in seizure frequency, and 18% achieved complete seizure freedom following 24-month intervention. However, we remain cautious when interpreting the findings due to small sample size and lack of a control group, limiting the study’s strength. Previous studies suggest that BCAAs could upregulate glutamate decarboxylase and subsequently increase GABA [42]. BCAAs could also modulate serotonin and dopamine synthesis and further influence brain excitatory/inhibitory balance by reducing aromatic amino acid uptake [43,44]. However, current evidence remains preliminary and limited to support BCAAs for epilepsy.

A recent case report showed that dietary supplementation of MCTs remarkably reduce seizure [45], suggesting that more ketogenic MCTs may offer more benefits than traditional vegetable oils used in KD. Two trials followed this direction and examined the effects of MCTs on epilepsy [28,30]. One RCT found that 12-month MCT-based KD was not superior to classical vegetable oil-based KD, though both diets significantly reduced seizure in children with DRE [30]. The other trial investigated the effects of MCTs supplementation on a regular diet and reported a 42% reduction in seizure frequency [28]. However, this trial was at a high risk of bias due to its a small sample size (*n* = 9) and a lack of a control group (pre-post design). Given this limitation, we find the evidence supporting MCTs for epilepsy is very week and inconclusive.

In addition to MCTs, olive oil, a component of Mediterranean diet has been investigated for its potential in epilepsy management [24]. Olive oil is rich in mono glyceride and has been widely studies for its antioxidant and anti-inflammatory activities [46,47]. The Guzel trial showed a very high respondent rate to olive-oil based KD: 83% of participants (389 children) experienced a >50% reduction in seizure frequency after 12-month dietary intervention. While the findings results are promising, the study design presents a high risk of bias, and further validation through a rigorous RCT is necessary to establish the efficacy of an olive oil-based KD diet in epilepsy management. Other than alternative modifications of KD, dietary supplementation of bioactive components have also been explored. Both Gómez-Eguílaz 2018 [11] and Shariatmadari 2024 [26] trials demonstrated that supplementation of probiotics and synbiotic significantly reduced seizure frequency and improved quality of life of epilepsy patients [11,26]. The probiotic approach targets gut microbiota which is believed to play a role in brain function and neurological disorders including epilepsy [32,48]. Certain gut bacteria have been known for their capability to produce or modulate neurotransmitters such as GABA, glutamate, and serotonin, which are involved in neuronal excitability and seizure thresholds [49]. Though both the trials suggest a potential of probiotics or synbiotic for epilepsy management, the current evidence is preliminary and with high risk of bias as both trials lacked placebo control. Furthermore, the trials used different probiotic/synbiotic formulations, making direct comparisons and definitive conclusions challenging.

The progression of dietary interventions for epilepsy beyond KD is marked by a shift towards exploring modifications to improve KD, and investigating alternative dietary strategies based on emerging understandings of epilepsy pathophysiology (MCT, probiotics, gut–brain axis, glutamate, etc.). Our synthesis reveals that while preliminary signals exist, most studies are small, open-label, and uncontrolled, which significantly limits their certainty. A significant methodological concern identified across the 8 studies is the high risk of bias in NRSIs. In the field of epilepsy, the “placebo effect” can account for up to a 20–30% reduction in seizure frequency in clinical trials. Therefore, the responder rates reported in uncontrolled studies must be interpreted with extreme caution until validated against a placebo in a blinded RCT. The only RCT in this review that compared a restrictive diet (low glutamate) to a control group yielded a negative result, highlighting the risk of overstating efficacy based on uncontrolled pilot data. Furthermore, there is a clear imbalance in the evidence. The largest study (Guzel 2019 [24]) focuses on a modification of the established ketogenic diet, where the sample synbiotics are represented by very small cohorts (*n* < 45). This disparity makes broad clinical conclusions difficult. Future research should prioritize rigorous RCTs with well-defined protocols and strong mechanistic rationales to determine the true clinical value of these alternative dietary strategies.

## 5. Interpretation of the Results

The findings of this review indicate a significant gap between the strong mechanistic rationale for alternative diets—such as the gut–brain axis for probiotics or GABA modulation for BCAAs—and the actual clinical evidence available to date. The high responder rates seen in uncontrolled trials (notably olive oil-based KDs) likely reflect a combination of true biological signal and the substantial placebo effect characteristic of epilepsy research. Consequently, these interventions should be interpreted as promising experimental targets rather than established clinical tools. The negative result from the only blinded RCT on glutamate restriction serves as a critical caution against adopting alternative diets based solely on mechanistic theory.

## 6. Conclusions

The clinical evidence for emerging dietary interventions beyond standard KD and fish oil supplementation is preliminary and of low certainty. While pilot data for olive oil-based KDs and probiotics are encouraging, they are currently insufficient to support routine clinical use. The negative result from the only RCT on glutamate restriction serves as a reminder that mechanistic theory requires rigorous validation. Future research must prioritize blinded RCTs that focus on specific patient sub-populations.

## Figures and Tables

**Table 1:** Characteristics of Enrolled Studies.

Study ID	Setting and Study Design	Intervention Details	Duration (Months)	Sample Size (N)	Gender (%Male)	Age
Evangeliou et al., 2009 [31]	Single-arm, pre-post intervention	BCAA powder (up to 20 g/d) + KD	6–24	17	N/A	2–7
Neal et al., 2009 [30]	Randomized trial	MCT-based ketogenic diet	3, 6, 12	145	52.40%	2–16
Guzel et al., 2019 [24]	Single-center, prospective study	Olive oil-based KD	1–12	389	51.90%	0.5–18
Gómez-Eguílaz et al., 2018 [11]	Single-arm, pre-post intervention	Probiotics (8 species, 4 × 10^11^/d)	4	45	53.30%	≥18
Shariatmadari et al., 2024 [26]	Pre-post quasi-experimental study	Synbiotics	2	30	60%	1–15
Rasmussen et al., 2023 [28]	Single-center open-label intervention	Supplemental MCT oil to regular diet	3	9	33.30%	24–63
Bashiri et al., 2016 [29]	Single-arm, pre-post intervention	Gluten-Free Diet	5	7	57%	26–38
Sarlo et al., 2023 [27]	Non-blinded, parallel, randomized clinical trial	Low glutamate diet	1	33	54.50%	2–21

Note: BCAA: branched amino acids, KD: ketogenic diet, AEDs: antiepileptic drugs, MCT: Medium-chain triglyceride.

**Table 2:** Individual Study Synthesis of Results.

Study ID	Intervention	Primary Outcomes	Synthesis of Results
Evangeliou et al., 2009 [31]	BCAA + KD	Seizure reduction	18% (3/17) seizure-free; 29% (5/17) had 50–90% seizure reduction vs. KD baseline.
Neal et al., 2009 [30]	MCT + KD	Seizure frequency	No significant differences vs. classical KD (*p* > 0.05 at 3, 6, or 12 months).
Guzel et al., 2019 [24]	Olive oil-KD	Responder rates (≥50% seizure reduction)	83.1% responder rate at 12 months; 43.1% seizure-free
Gómez-Eguílaz et al., 2018 [11]	Probiotics	Responder rate (≥50% seizure reduction)	28.9% (13/15) achieved ≥50% seizure reduction
Shariatmadari et al., 2024 [26]	Synbiotics	Seizure frequency	Significant decrease (Pre: 15.83, Post: 12.73, *p* = 0.001).
Rasmussen et al., 2023 [28]	MCT	Seizure frequency	42% reduction in seizures (*p* < 0.0001).
Bashiri et al., 2016 [29]	GFD (for Celiac)	Seizure freedom	86% (6/7) achieved seizure freedom.
Sarlo et al., 2023 [27]	Low Glutamate	Seizure frequency	Non-Seizure improvements (*p* = 0.57).

## Data Availability

Data sharing is not applicable to this article as no new data were created or analyzed in this study.

## Supplementary Materials

The following supporting information can be downloaded at: https://www.mdpi.com/article/10.3390/neurolint18010009/s1, Table S1: PRISMA 2020 Checklist.
