# Supplementary material for: Exploratory Dietary Approaches for Drug-Resistant Epilepsy Beyond Standard Ketogenic Diet and Fish Oil: A Systematic Review of Preliminary Clinical Evidence
Source: Neurol Int. 2026 Jan 4;18(1):9. doi: 10.3390/neurolint18010009 (PMC12844633; doi:10.3390/neurolint18010009)
Supplement: Supplementary file 1 [file neurolint-18-00009-s001.zip › neurolint-4042072-supplementary.pdf]

**Exploratory Dietary Approaches for Drug-Resistant Epilepsy Beyond Standard Ketogenic Diet and Fish Oil: A Systematic Review of Preliminary Clinical Evidence**

**Xianghong Meng and Kequan Zhou**

**Table S1: PRISMA 2020 Checklist**

| Section and Topic | Item # | Checklist Item | Reported (Yes/No) | Location in Manuscript |
|---|---|---|---|---|
| **TITLE** | | | | |
| Title | 1 | Identify the report as a systematic review. | **Yes** | Title Page |
| **ABSTRACT** | | | | |
| Abstract | 2 | See the PRISMA 2020 for Abstracts checklist. | **Yes** | Abstract |
| **INTRODUCTION** | | | | |
| Rationale | 3 | Describe the rationale for the review in the context of existing knowledge. | **Yes** | Section 1 |
| Objectives | 4 | Provide an explicit statement of the objective(s) or question(s) the review addresses. | **Yes** | Section 1 |
| **METHODS** | | | | |
| Eligibility criteria | 5 | Specify the inclusion and exclusion criteria for the review and how studies were grouped for the syntheses. | **Yes** | Section 2.2 |
| Information sources | 6 | Specify all databases, registers, websites, and other sources searched. | **Yes** | Section 2.1 |
| Search strategy | 7 | Present the full search strategies for all databases, registers and websites, including any filters used. | **Yes** | Section 2.1 |
| Selection process | 8 | Specify the methods used to decide whether a study met the inclusion criteria of the review. | **Yes** | Section 2.2 |
| Data collection process | 9 | Specify the methods used to collect data from reports. | **Yes** | Section 2.3 |
| Data items | 10a | List and define all outcomes for which data were sought. | **Yes** | Section 2.3 |

| Section and Topic | Item # | Checklist Item | Reported (Yes/No) | Location in Manuscript |
|---|---|---|---|---|
| Study risk of bias assessment | 11 | Specify the methods used to assess risk of bias in the included studies. | **Yes** | Section 2.4 |
| Synthesis methods | 13a | Describe the processes used to decide which studies were eligible for each synthesis. | **Yes** | Section 2.5 |
| | 13d | Describe any methods used to explore associations between study characteristics and outcomes. | **Yes** | Section 2.5 |
| **RESULTS** | | | | |
| Study selection | 16a | Describe the results of the search and selection process, from the number of records identified in the search to the number of studies included in the review. | **Yes** | Section 3.1 & Figure 1 |
| Study characteristics | 17 | Cite each included study and present its characteristics. | **Yes** | Table 1 |
| Risk of bias in studies | 18 | Present assessments of risk of bias for each included study. | **Yes** | Section 3.1 & 3.2 |
| Results of individual studies | 19 | For all outcomes presented, present for each study: summary statistics and an estimate of effect. | **Yes** | Table 2 |
| **DISCUSSION** | | | | |
| Discussion | 23a | Provide a general interpretation of the results in the context of other evidence. | **Yes** | Section 4 |
| | 23c | Discuss any limitations of the review processes used. | **Yes** | Section 4 |
| | 23d | Discuss implications of the results for practice, policy, and future research. | **Yes** | Section 4 & 5 |
| **OTHER INFO** | | | | |
| Registration and protocol | 24a | Provide registration information for the review, or state that the review was not registered. | **Yes** | Section 2.1 |
| Funding | 25 | Describe sources of financial or non-financial support for the review. | **Yes** | Acknowledgments |
| Conflict of interest | 26 | Declare any competing interests of review authors. | **Yes** | Conflicts of Interest Section |
